# Unraveling Gender Differences in Obsessive-Compulsive Disorder: A Focus on Key Micronutrients

**DOI:** 10.7759/cureus.79667

**Published:** 2025-02-25

**Authors:** Sultana Algin, Mohammad Waliul Hasnat Sajib, Sumaiya Nausheen Ahmed, Md Raihan Siddique, Md Munim Reza, Nusrat Jahan Tanzilla, Tanbir Ahmed, Md Kamrul Islam, Pratiksha Patel, Mainul Haque

**Affiliations:** 1 Department of Psychiatry, Bangabandhu Sheikh Mujib Medical University, Dhaka, BGD; 2 Department of Psychiatry, Tangail Medical College, Tangail, BGD; 3 Department of Psychiatry, Universal Medical College and Hospital Ltd., Dhaka, BGD; 4 Department of Psychiatry, Delta Medical College, Dhaka, BGD; 5 Department of Psychiatry, Enam Medical College and Hospital, Savar, BGD; 6 Department of Periodontology, Karnavati School of Dentistry, Karnavati University, Gandhinagar, IND; 7 Department of Pharmacology and Therapeutics, National Defence University of Malaysia, Kuala Lumpur, MYS; 8 Department of Research, Karnavati School of Dentistry, Karnavati University, Gandhinagar, IND

**Keywords:** biomarkers of ocd, folic acid, homocysteine, neuroinflammation, nutrition-related psychiatric comorbidities, obsessive-compulsive neurosis, psychiatric biochemistry, sex difference, vitamin b12, yale-brown obsessive-compulsive scale

## Abstract

Introduction

Obsessive-compulsive disorder (OCD) is a persistent psychiatric condition that causes significant clinical and functional impairments. Recent research suggests a link between deficiencies in micronutrients, particularly vitamin B_12_, folic acid, and elevated homocysteine, and the development of OCD. This study investigates the blood levels of these micronutrients and their relationship to OCD severity, with an emphasis on potential gender differences.

Methods

This cross-sectional study included 300 drug-free OCD patients. Serum levels of vitamin B_12_, folic acid, and homocysteine were measured using established biochemical methods. The Yale-Brown Obsessive Compulsive Scale (Y-BOCS) was used to assess clinical severity. Data were examined to determine relationships between micronutrient levels and OCD severity and differences between male and female patients.

Results

This study found that women had higher levels of vitamin B_12 _(405.3 ± 15.1 vs. 360.4 ± 14.3) and folic acid (7.18 ± 0.27 vs. 5.76 ± 0.25) but lower levels of homocysteine (9.35 ± 0.64 vs. 14.4 ± 0.60) compared to men. Higher folic acid levels were linked to study participants having higher levels of education (at a college or university, where subjects are studied at an advanced level) compared to those with primary-level education. Lower vitamin B_12 _levels were linked to family mental health history and noncommunicable diseases. Women exhibited lower levels of substance use but higher rates of self-harm and suicide attempts. Elevated homocysteine levels were linked to longer illness duration and more severe OCD symptoms.

Conclusion

These findings suggest that imbalances in micronutrients, particularly vitamin B_12_, folic acid, and homocysteine, may contribute to OCD severity and treatment resistance. Gender-specific variations in micronutrient levels could provide valuable insights into personalized OCD therapy choices. Further longitudinal studies are needed to understand these relationships and their potential as therapeutic targets.

## Introduction

Obsessive-compulsive disorder (OCD) is a prevalent chronic, debilitating psychiatric condition affecting around 1%-3% of the global population [1-3]. The Epidemiological Catchment Area Survey ranks OCD as the fourth most common psychiatric disorder, while the early burden of disease research places it as the 10th most incapacitating medical disorder overall [2-4]. According to the National Survey Bangladesh 2018-2019, the prevalence of OCD among adults [5,6] and children [7] was 0.7% and 2%, respectively.

There are no established medical diagnostic biomarkers that can definitively detect OCD [8-10]. Research on micronutrient deficiencies, such as vitamin B_12_, folic acid, and homocysteine, and their potential link to OCD development, especially in low- and middle-income countries, remains limited [11]. One systematic review and meta-analysis found decreased vitamin B_12_ levels, increased homocysteine levels, and normal folate levels in patients with OCD [12]. Several studies have also reported hyperhomocysteinemia in various psychiatric disorders, including mood disorders, schizophrenia, depression, and bipolar disorder [13-18]. Some earlier studies found that a subset of OCD patients exhibited folate deficiency [11,19,20], though no statistically significant difference in folate levels was observed between those with and without OCD [12].

Impaired methylation and monoamine metabolism have also been observed [21-23], potentially serving as significant biological markers for OCD [12,24,25]. Vitamin B_12_ deficiency is associated with low-grade neuroinflammation and is present in around 20% of cases [26-28]. Furthermore, vitamin B_12 _deficiency can also trigger a range of neuropsychiatric conditions, e.g., acute psychosis, anxiety, apathy, cognitive changes, compromised memory, delirium, delusions, dementia, depression, episodes of disorientation, irritability, mania, paranoid state, schizophrenia, and seldom suicidal ideation [29-32]. Several studies have shown that low serum folate levels are linked to a higher risk of developing depression [33-35]. Folate-deficient individuals, especially those with “treatment-resistant” depression, are more likely to experience severe affective disorders and respond poorly to conventional treatments [33,36]. The Mayo Clinic reported that deficiencies in vitamin B_12 _and other B vitamin (such as B_6_) are associated with depression [37]. Moreover, research on vitamin B_12_ supplementation has yielded varied and inconclusive results regarding its potential to reduce the development of depression [37,38].

Women typically experience a later onset of OCD symptoms than men, although men are more frequently affected during childhood and adolescence [39,40]. The average age of onset for OCD is between 19.5 and 20 years [41,42], with men generally showing symptoms somewhat earlier than women [43,44]. OCD is associated with a lower quality of life and substantial social and occupational impairment [45,46]. Suicidal thoughts are reported by 36%-63.5% of OCD patients [47-49] and suicidal attempts by 10%-16.5% [50,51]. Depressive disorders are commonly co-occurring with OCD, with lifetime prevalence rates as high as 48% [52,53]. In their paper, Devi et al. reported that the prevalence of OCD among schizophrenic patients ranges from 0% to 64% [54]. A recent study by Ahn-Robbins et al. revealed that among patients with "schizophrenia, schizoaffective disorder, or bipolar disorder," obsessive-compulsive symptoms (OCS) and OCD developed 24% and 11.9%, respectively [55]. Around 20%-30% of OCD patients concomitantly develop tics, while 22%-44% of patients with tics concurrently develop OCD [56]. According to DSM-5, a novel subdivision of OCD (called tic-associated OCD) affects 10%-40% of childhood OCD [57-59].

It was postulated in earlier research that monoamine neurotransmitter deficiency, especially serotonin, plays a significant role in the pathogenesis of OCD [60,61]. Vitamin B_12_ and folic acid are crucial for methylation processes involved in neurotransmitter synthesis [62]. Many OCD patients, except for those with well-defined OCD symptoms, often go undiagnosed in clinical settings [63-65], with micronutrient deficiencies frequently overlooked in these cases [63]. Deficiencies in vitamin B_12_, folic acid, and elevated homocysteine levels are commonly associated with treatment-resistant OCD [11,12]. Thus, this study aims to accurately measure vitamin B_12_, folic acid, and homocysteine levels to determine whether variations in these micronutrients contribute to the development and/or progression of OCD.

Problem statement of the paper

OCD is a chronic mental health condition characterized by intrusive thoughts and repetitive behaviors [66]. Despite significant advancements in treatment, the exact etiology and factors influencing the progression of OCD remain unclear [67]. One area of growing interest is the potential role of micronutrients, such as vitamin B_12_, folic acid, and homocysteine, in the pathophysiology of OCD [11,12,20,68]. While multiple studies have suggested that abnormalities in these micronutrients may be linked to various psychiatric disorders, few have explicitly focused on their role in OCD [69-71]. Assessing these biochemical factors in conjunction with clinical and demographic characteristics is critical for understanding their potential contribution to the development and progression of OCD [72-74].

Objectives of the study

This cross-sectional study aimed to assess serum levels of vitamin B_12_, folic acid, and homocysteine in patients with OCD and explore the correlation between serum micronutrient levels (vitamin B_12_, folic acid, homocysteine) and OCD symptom severity, as measured by the Yale-Brown Obsessive-Compulsive Scale (Y-BOCS) and the Dimensional Obsessive-Compulsive Scale (DOCS). The study also sought to identify any sociodemographic components (e.g., occupation, marital status, family history, gender) associated with abnormal levels of vitamin B_12_, folic acid, and homocysteine in OCD patients. Additionally, the study aimed to determine whether metabolic abnormalities in micronutrients (vitamin B_12_, folic acid, homocysteine) contribute to the etiopathogenesis and/or progression of OCD. These findings could provide insights into the potential link between metabolic and dietary abnormalities and the clinical characteristics of OCD, ultimately informing future treatment approaches.

## Materials and methods

The study was carried out in two phases. The first phase was a cross-sectional descriptive study conducted at the Department of Psychiatry, Bangabandhu Sheikh Mujib Medical University (BSMMU), Dhaka, from April 2019 to October 2023 (cases from 2020 to 2022 could not be included due to the COVID-19 pandemic). This study was approved by the Institutional Review Board (IRB) at BSMMU, Dhaka, Bangladesh. Written informed consent and demographic data were collected from the participants using a semi-structured questionnaire. We developed a sociodemographic and clinical questionnaire based on previous publications, incorporating variables such as age, sex, education, occupation, residence, religion, economic status, birth order and onset, family history of the disorder, comorbidity, and duration of illness [75-77]. The questions were adapted from various studies and modified by a BSMMU psychiatrist to align with Bangladesh's social context [78-81]. Triangulation methods were used to validate the survey data to enhance the validity and reliability of the findings [82].

Study subject selection strategy

The inclusion criteria were as follows: (1) age between 18 and 60 years, (2) patient diagnosed with OCD by a psychiatrist using the Diagnostic and Statistical Manual of Mental Disorders, Fifth Edition (DSM-5), and (3) those who did not receive psychological therapy or psychiatric medication in the four weeks prior to study enrolment [11,19,20,83].

The exclusion criteria were as follows: (1) severe acute psychiatric disorders, (2) serious physical ailments including liver disease, kidney disease, or organic brain abnormalities (such as dementia, ischemic and hemorrhagic strokes), (3) a 12-month history of active alcohol misuse, (4) disorders such as pernicious anemia or those affecting the absorption of vitamin B_12 _such as gastric surgery, (5) use of multivitamin supplements, folic acid, or vitamin B_12_ in the past 12 months, (6) strict vegetarians, pregnant ladies, and nursing mothers, and (7) Intellectual disability (formerly referred to as mental retardation) [11,19,20,83].

**Figure 1:**
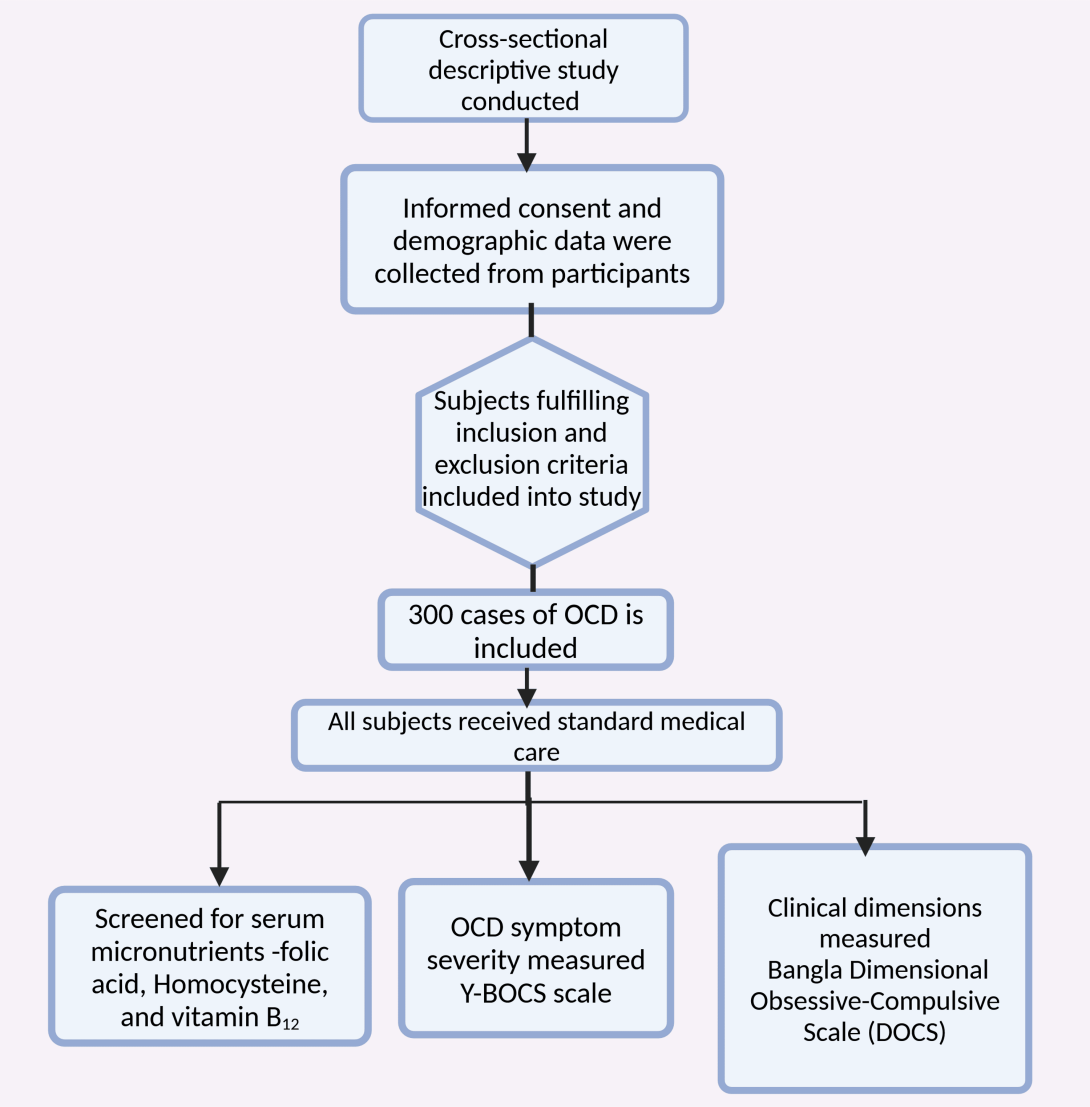
Flowchart illustrating the methodology of the study. Note: This figure was drawn using the premium version of BioRender (https://www.biorender.com/) [86], accessed on January 24, with agreement license number UA27TU1MRC. Illustration Credit: Pratiksha Patel.

Three hundred cases of OCD were selected for this study from the outpatient, inpatient, and OCD clinic departments at BSMMU Hospital. Convenience sampling was used to select the cases. All participants received standard medical care throughout the study (Figure 1). Serum micronutrient levels (folic acid, homocysteine, and vitamin B_^12^_) were measured at the biochemistry laboratory of BSMMU. After that, OCD symptom severity was assessed using the Bangla version of the Y-BOCS [84]. The results were interpreted based on the total score [85]: 0-7, subclinical; 8-15, mild; 16-23, moderate; 24-31, severe; and 32-40, extreme.

The Bangla DOCS was used for clinical dimensions (English translation, Appendix 1) [87]. This 20-item self-report tool is designed to assess the severity of OCD symptoms across four dimensions: (a) contamination, (b) responsibility for harm and mistakes, (c) incompleteness or symmetry, and (d) unacceptable (taboo) thoughts. Each subscale includes a general description and examples of obsessions and compulsions related to that dimension. Respondents rate their symptoms from the past month on a scale from 0 (no symptoms) to 4 (extreme symptoms) based on five criteria: time spent on symptoms, avoidance behavior, distress, functional interference, and difficulty controlling the symptoms. The five items in each subscale are summed to give a subscale score (range: 0-20), and the four subscale scores can be summed for a total DOCS score (range: 0-80). This method assesses the severity of a patient's symptoms rather than predefined symptom criteria [88]. Lastly, insight into the patient's condition was measured. The cutoff values for identifying micronutrient imbalances are shown in Table 1.

**Table 1:** Abnormalities in micronutrients identified using cutoff values. Source: [11].

Name	Cutoff values
Serum vitamin B_12_	<200 pg/mL (vitamin B_12_ deficiency)
Serum folate level	<3 ng/mL (folate deficiency)
Plasma homocysteine	>14.0 μmol/L (hyperhomocysteinemia)

Blood sample collection

Venous blood samples were collected under aseptic conditions after an overnight fast. A total of 6 mL was drawn: 3-4 mL in a plain (red-top) tube for vitamin B_^12^_ and folic acid and 2-3 mL in an ethylenediaminetetraacetic acid (EDTA) (purple-top) tube for homocysteine. Samples were centrifuged at 3000 rpm for five minutes within 30 minutes, and serum and plasma were separated and stored in polythene tubes [89,90].

Biochemical analysis

Serum vitamin B_12 _was assessed using a solid-phase, competitive chemiluminescent immunoassay [91]. The folic acid concentration was measured via a competitive, ligand-labeled chemiluminescent assay [92]. Plasma homocysteine was quantified using a competitive chemiluminescent enzyme immunoassay [93]. All assays were performed on the Immulite 1000 chemiluminescent analyzer (Siemens Healthineers, Erlangen, Germany) at the Department of Biochemistry and Molecular Biology, BSMMU, Dhaka.

Ethical approval

All ethical issues were considered before the study commenced. Ethical approval was obtained from the IRB of Bangabandhu Sheikh Mujib Medical University (reference no. BSMMU/2019/4141, dated 23-04-2019). The COVID-19 pandemic delayed the start of our research, which was deferred for more than two years. Written informed consent was obtained from all participants, and the research plan, including future publication, was explained. All other issues relating to research were maintained and given due importance.

Statistical analysis plan

The descriptive characteristics of OCD patients and their clinical symptoms were analyzed and presented as mean ± SD for continuous variables and frequency with percentages for categorical variables. We used a univariate regression model to examine the mean differences in vitamin B_12_, serum folic acid, and homocysteine levels between men and women. A multiple regression model was employed to assess the effects of predictors, including demographic characteristics and OCD clinical symptoms, on vitamin B_12_, serum folic acid, and homocysteine levels. Ordinal logistic regression was used to evaluate the risk of OCD clinical symptoms among women and men for binary outcomes, while ordinal outcomes were analyzed using multinomial logistic regression. The logistic regression models were adjusted for age, sex, marital status, education level, occupation, residence, family pattern, and birth order. All statistical analyses were conducted using Stata, Version 15 (StataCorp LLC, College Station, TX, United States), and figures were generated using GraphPad Prism 8.3.2 (Dotmatics, Boston, MA, United States). A p-value of <0.05 was considered statistically significant.

## Results

Table 2 provides a detailed overview of the demographic, clinical, and psychosocial characteristics of 300 OCD patients. The mean age was 27.3 ± 9.08 years, with a slight male predominance (53%, n = 159). Most participants were unmarried (55%, n = 165), had secondary education (43.3%, n = 130), and resided in urban areas (69%, n = 207). Regarding occupation, students formed the largest group (42.7%, n = 128), followed by housewives (21.7%, n = 65) and employed individuals (20.7%, n = 62). Most lived in nuclear families (61.7%, n = 185) and were Muslim (92.3%, n = 277). Birth order analysis showed that 36.3% (n = 109) were first-born, while 42% (126) were in other positions. Thirty-six percent (n = 108) reported a family history of mental illness, and 22.7% (n = 68) had physical comorbidities, with hypothyroidism (4.67%, n = 14) and skin disorders (4%, n = 12) being the most common. Psychiatric comorbidities were observed in 33% (n = 99), with depressive disorder (11.7%, n = 35) and generalized anxiety disorder (12.3%, n = 37) being the most frequent. Substance abuse history was reported by 15.3% (n = 46), primarily involving cannabis (3.67%, n = 11), nicotine (3.33%, n = 10), and alcohol (3%, n = 9). Additionally, 10.3% had a history of deliberate self-harm, and 8% had attempted suicide. OCD onset was juvenile in 39.3% (n = 118) and adult in 60.7% (n = 182), with most patients having an illness duration of less than five years (63%, n = 189). Symptom severity, measured using Y-BOCS scores, revealed that 39.7% (n = 118) had severe OCD, 14.3% (n = 43) had extreme OCD, and only 0.67% (n = 2) had subclinical symptoms. Insight levels varied, with 47% (n = 141) demonstrating good insight, 19.7% (n = 59) poor insight, and 1.33% (n = 4) lacking insight.

**Table 2:** Demographic characteristics of patients with obsessive-compulsive disorder. Note: Data were presented as mean ± SD or number with percent in parentheses. H/O: history of, GAD: generalized anxiety disorder, Y-BOCS: Yale-Brown Obsessive Compulsive Scale.

Independent observations	Response (n = 300)
Age, years	27.3 ± 9.08
Sex	
Male	159 (53.0%)
Female	141 (47.0%)
Marital status	
Unmarried	165 (55.0%)
Married	125 (41.7%)
Widowed/separated/divorced	10 (3.33%)
Education qualification	
Illiterate	9 (3.00%)
Primary	40 (13.3%)
Secondary	130 (43.3%)
Graduate	95 (31.7%)
Postgraduate	26 (8.67%)
Occupation	
Unemployed	34 (11.3%)
Student	128 (42.7%)
Housewife	65 (21.7%)
Service	62 (20.7%)
Others	11 (3.66%)
Residence	
Rural	93 (31.0%)
Urban	207 (69.0%)
Religion	
Muslim	277 (92.3%)
Hindu	23 (7.67%)
Family pattern	
Nuclear	185 (61.7%)
Joined	106 (35.3%)
Extended	9 (3.00%)
Birth order	
First	109 (36.3%)
Last	65 (21.7%)
Others	126 (42.0%)
Family H/O mental illness	108 (36.0%)
Comorbidity	
Hypothyroidism	14 (4.67%)
Diabetes	7 (2.33%)
Hypertension	7 (2.33%)
Skin disorder	12 (4.00%)
Bronchial asthma	10 (3.33%)
Others	18 (6.00%)
None	232 (77.3%)
Comorbid psychiatric illness	
Panic disorder	6 (2.00%)
Social phobia	3 (1.00%)
Agoraphobia	1 (0.33%)
Depressive disorder	35 (11.7%)
GAD	37 (12.3%)
Personality disorder	9 (3.00%)
Psychotic disorder	2 (0.67%)
Others	6 (2.00%)
None	201 (67.0%)
H/O substance abuse	
Sedatives	2 (0.67%)
Cannabis	11 (3.67%)
Alcohol	9 (3.00%)
Nicotine	10 (3.33%)
Energy drink	1 (0.33%)
Others	13 (4.33%)
None	254 (84.7%)
H/O deliberate self-harm	31 (10.3%)
H/O suicide attempts	24 (8.00%)
Onset	
Juvenile (before 18 years of age)	118 (39.3%)
Adult	182 (60.7%)
Duration of illness	
<5 years	189 (63.0%)
6-10 years	63 (21.0%)
11-15 years	32 (10.7%)
16-20 years	8 (2.67%)
>20 years	8 (2.67%)
Y-BOCS score category	
Subclinical (0-7)	2 (0.67%)
Mild (8-15)	43 (14.3%)
Moderate (16-23)	93 (31.0%)
Severe (24-31)	119 (39.7%)
Extreme (32-40)	43 (14.3%)
Insight levels	
Excellent	43 (14.3%)
Good	141( 47.0%)
Fair	53 (17.7%)
Poor	59 (19.7%)
Lack of insight	4 (1.33%)

**Figure 2:**
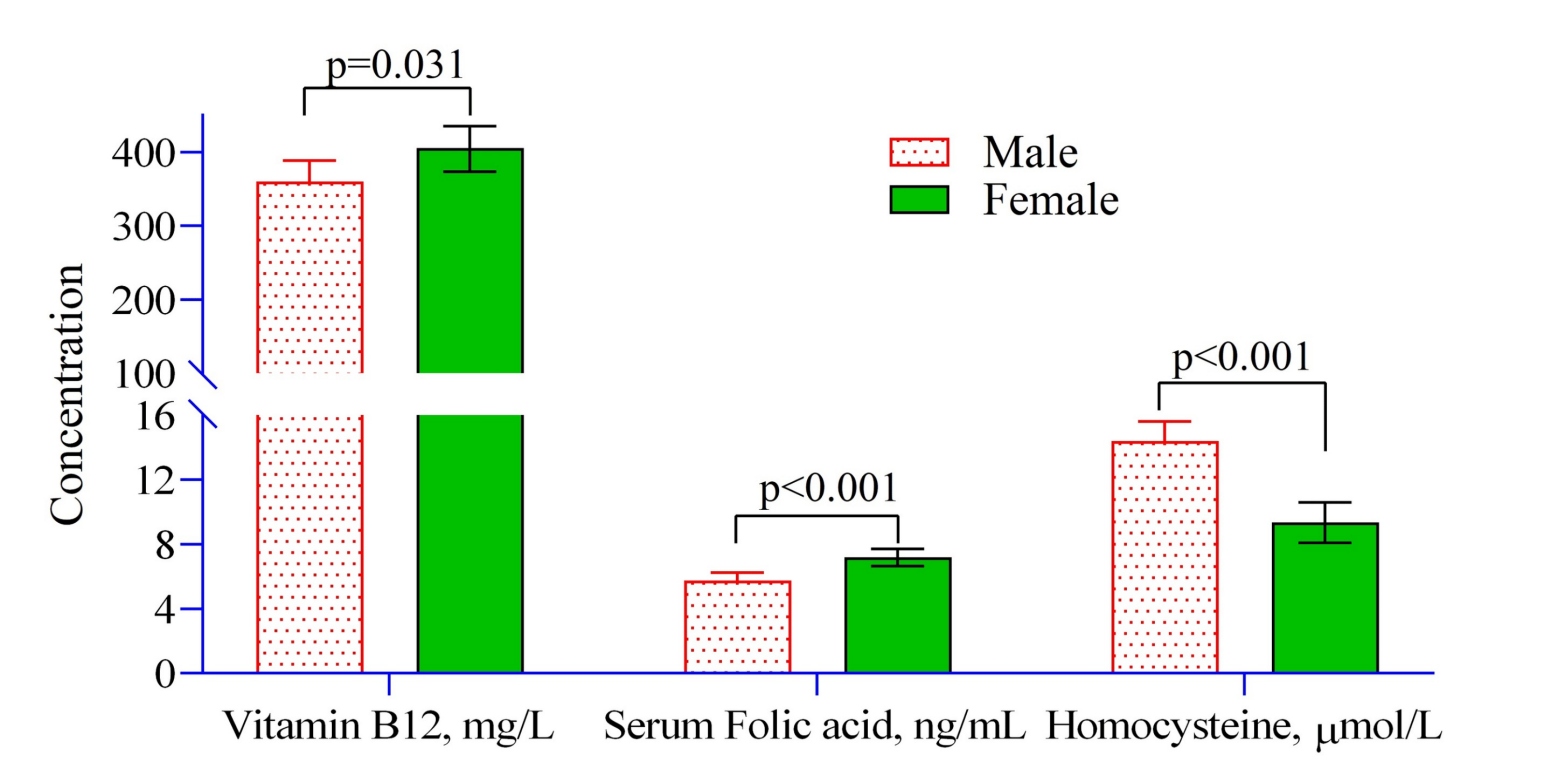
Mean differences in vitamin B12, serum folic acid, and homocysteine levels between men and women with obsessive-compulsive disorder. Note: Univariate regression model was used to estimate the p-value. Illustration Credit: Md. Ahsanul Haq.

The analysis reveals significant gender differences in serum vitamin B_12_, folic acid, and blood homocysteine levels. Women had higher mean serum vitamin B_12_ levels (405.301 ± 15.135, 95% CI 375.516-435.086) compared to men (360.356 ± 14.252, 95% CI 332.308-388.405) (Figure 2). Similarly, serum folic acid levels were higher in women (7.181 ± 0.268, 95% CI 6.653-7.709) than in men (5.755 ± 0.253, 95% CI 5.258-6.252). In contrast, blood homocysteine levels were significantly lower in women (9.352 ± 0.640, 95% CI 8.093-10.611) compared to men (14.424 ± 0.603, 95% CI 13.238-15.610) (Figure 2). These findings suggest potential gender-specific metabolic or nutritional differences among OCD patients.

While examining the association between independent characteristics and OCD-related symptoms with vitamin B_12_ levels, women exhibited significantly higher vitamin B_12_ concentrations (96.1 mg/L) compared to men (p < 0.001). In contrast, homemakers had statistically considerably lower vitamin B_12_ levels (97.0 mg/L; 95% CI -183.4, -81.3; p = 0.028) compared to unemployed participants (Table 3).

**Table 3:** Association between independent and OCD disease-related markers with vitamin B12. Note: ^$^Hypothyroidism, diabetes, hypertension, and bronchial asthma.  ^&^Panic disorder, social phobia, agoraphobia, personality disorder, and Psychotic disorder. Multiple regression models were used to estimate the p-value. H/O: history of, NCD: noncommunicable disease, GAD: generalized anxiety disorder, Y-BOCS: Yale-Brown Obsessive Compulsive Scale.

Vitamin B_12_ (n = 300)			
Independent observations		β-coff (95% CI)	p-value
Age, years	N/A	0.68 (-2.65, 4.01)	0.689
Sex	Male	Ref. (0)	N/A
	Female	96.1 (44.4, 147.8)	<0.001
Educational qualification	Primary	Ref. (0)	N/A
	Secondary	7.59 (-54.3, 69.5)	0.810
	Graduate	14.2 (-54.6, 83.1)	0.684
	Postgraduate	-36.3 (-129.3, 56.6)	0.442
Occupation	Unemployed	Ref. (0)	N/A
	Student	-31.5 (-108.3, 45.3)	0.420
	Housewife	-97.0 (-183.4, -81.3)	0.028
	Service	-34.3 (-184.9, 116.4)	0.655
Residence	Rural	Ref. (0)	N/A
	Urban	-5.32 (-52.6, 41.9)	0.825
Family pattern	Nuclear	Ref. (0)	N/A
	Joined	26.2 (-19.3, 71.7)	0.258
	Extended	104.0 (-21.0, 119.1)	0.103
Birth order	First	Ref. (0)	N/A
	Last	-71.4 (-130.3, -12.6)	0.018
	Others	-69.4 (-118.3, -20.6)	0.006
Family H/O mental illness	No	Ref. (0)	N/A
	Yes	-47.7 (-93.7, -1.72)	0.042
Comorbidity	No comorbidity	Ref. (0)	N/A
	NCD^$^	-91.1 (-156.6, -25.6)	0.921
	Skin disorder	-56.6 (-164.9, 51.7)	0.423
	Others	0.12 (-92.5, 92.8)	0.998
Comorbid psychiatric illness	No psychiatric illness	Ref. (0)	N/A
	Depressive disorder	-0.35 (-68.2, 67.5)	0.921
	GAD	11.7 (-54.8, 78.1)	0.423
	Others^&^	0.12 (-92.5, 92.8)	0.998
H/O substance abuse	No substance use	Ref. (0)	N/A
	Substance use	-5.08 (-66.0, 55.9)	0.870
H/O deliberate self-harm	No	Ref. (0)	N/A
	Yes	50.3 (31.5, 132.2)	0.021
H/O suicide attempts	No	Ref. (0)	N/A
	Yes	-40.5 (0130.9, 49.8)	0.378
Onset	Juvenile (before 18 years of age)	Ref. (0)	N/A
	Adult	-20.7 (-74.9, 33.6)	0.454
Duration of illness	<5 years	Ref. (0)	N/A
	6-10 years	14.2 (-40.1, 68.6)	0.607
	11-15 years	13.8 (-58.4, 85.9)	0.707
	>16 years	011.7 (-119.0, 95.5)	0.829
Y-BOCS score category	Mild (8-15)	Ref. (0)	N/A
	Moderate (16-23)	-167 (-84.3, 50.9)	0.627
	Severe (24-31)	-50.1 (-117.6, 17.5)	0.146
	Extreme (32-40)	-40.1 (-122.4, 42.3)	0.339
Insight levels	Poor	Ref. (0)	N/A
	Excellent	-16.8 (-96.0, 62.4)	0.676
	Good	-29.2 (-87.9, 29.6)	0.329
	Fair	-57.5(-127.4, 12.4)	0.106

Birth order also showed a significant effect on vitamin B_12_ levels, with participants in the last birth order and other birth orders having significantly lower concentrations (71.4 mg/L (p = 0.018) and 69.4 mg/L (p=0.006), respectively) compared to first-born participants.

Participants with a family history of mental illness demonstrated lower vitamin B_12_ levels (47.7 mg/L, p = 0.042) (Table 3). Additionally, participants with noncommunicable diseases (NCDs) had significantly lower vitamin B_12_ levels (β = -91.1; 95% CI -156.6, -25.6; p = 0.007) compared to those without comorbidities.

Interestingly, participants with a history of deliberate self-harm exhibited elevated vitamin B_12_ levels (β = 50.3; 95% CI 31.5, 132.2; p = 0.021) (Table 3).

Like vitamin B_12_, serum folic acid was also higher among women (1.09 ng/mL, p = 0.023) than men. Educational level was significantly associated with increased folic acid levels, with participants having secondary, graduate, and postgraduate education showing levels of 1.52 ng/mL, 1.27 ng/mL, and 1.84 ng/mL, respectively, compared to those with primary-level education (Table 4). Housewives showed higher concentrations of folic acid (1.46 ng/mL; 95% CI = 0.001, 3.05; p = 0.049) compared to unemployed patients. No other significant observations were noted.

**Table 4:** Association between independent and OCD disease-related symptoms with serum folic acid. Note: ^$^Hypothyroidism, diabetes, hypertension, and bronchial asthma. ^&^Panic disorder, social phobia, agoraphobia, personality disorder, and psychotic disorder. A multiple regression model was used to estimate the p-value. H/O: history of, NCD: noncommunicable disease, GAD: generalized anxiety disorder, Y-BOCS: Yale-Brown Obsessive Compulsive Scale.

Folic acid (n = 300)			
Independent observations		β-coff (95% CI)	p-value
Age, years	N/A	0.01 (-0.05, 0.07)	0.811
Sex	Male	Ref. (0)	N/A
	Female	1.09 (0.15, 2.03)	0.023
Educational qualification	Primary	Ref. (0)	N/A
	Secondary	1.52 (0.40, 2.64)	0.008
	Graduate	1.27 (0.02, 2.51)	0.046
	Postgraduate	1.84 (0.16, 3.52)	0.027
Occupation	Unemployed	Ref. (0)	N/A
	Student	0.39 (-1.00, 1.78)	0.579
	Housewife	1.46 (0.001, 3.05)	0.049
	Service	0.69 (-0.72, 2.11)	0.337
	Others	2.38 (-1.02, 5.78)	0.169
Residence	Rural	Ref. (0)	N/A
	Urban	-0.46 (-1.31, 0.40)	0.292
Family pattern	Nuclear	Ref. (0)	N/A
	Joined	0.14 (-0.68, 0.97)	0.730
	Extended	-1.24 (-3.50, 1.02)	0.281
Birth order	First	Ref. (0)	N/A
	Last	-0.86 (-1.92, 0.21)	0.114
	Others	-0.17 (-1.06, 0.71)	0.697
Family H/O mental illness	No	Ref. (0)	N/A
	Yes	0.24 (-0.59, 1.07)	0.774
Comorbidity	No comorbidity	Ref. (0)	N/A
	NCD^$^	0.16 (-1.02, 1.35)	0.785
	Skin disorder	-0.59 (-2.55, 1.37)	0.553
	Others	0.58 (-1.09, 2.25)	0.496
Comorbid psychiatric illness	No psychiatric illness	Ref. (0)	N/A
	Depressive disorder	0.12 (-1.11, 1.34)	0.850
	GAD	0.58 (-0.62, 1.78)	0.344
	Others^&^	0.71 (-0.71, 2.13)	0.324
H/O substance abuse	No substance use	Ref. (0)	N/A
	Substance use	-0.58 (-1.68, 0.52)	0.300
H/O deliberate self-harm	No	Ref. (0)	N/A
	Yes	0.75 (-0.73, 2.23)	0.318
H/O suicide attempts	No	Ref. (0)	N/A
	Yes	-0.94 (-2.57, 0.69)	0.259
Onset	Juvenile (before 18 years of age)	Ref. (0)	N/A
	Adult	-0.52 (-1.50, 0.46)	0.293
Duration of illness	<5 years	Ref. (0)	N/A
	6-10 years	-0.49 (-1.47, 0.49)	0.328
	11-15 years	-0.93 (-2.23, 0.37)	0.162
	>16 years	-0.22 (-2.16, 1.72)	0.823
Y-BOCS score category	Mild (8-15)	Ref. (0)	N/A
	Moderate (16-23)	-0.20 (-1.43, 1.02)	0.742
	Severe (24-31)	-0.45 (-1.67, 0.77)	0.465
	Extreme (32-40)	-0.50 (-1.99, 0.98)	0.506
Insight levels	Poor	Ref. (0)	N/A
	Excellent	-0.35 (-1.78, 1.08)	0.629
	Good	0.25 (-0.81, 1.31)	0.640
	Fair	0.09 (-1.17, 1.35)	0.889

Homocysteine levels were significantly lower among women with OCD (β = -4.97; 95% CI -7.20, -2.74; p < 0.001) compared to men. In contrast, patients under the "other occupations" group had significantly higher homocysteine levels by 8.11 units compared to unemployed patients (p = 0.014). A strong positive association was observed with the duration of symptoms. Patients with symptoms lasting more than 16 years had significantly elevated homocysteine levels (β = 4.32; 95% CI 0.29, 8.92; p = 0.042) compared to those with less than five years of symptom duration. Similarly, the Y-BOCS score showed a significant positive association, with the extreme score group displaying higher homocysteine levels (β = 4.04; 95% CI 0.50, 7.58; p = 0.026) (Table 5).

**Table 5:** Association between independent and OCD disease-related markers with homocysteine. Note: ^$^Hypothyroidism, diabetes, hypertension, and bronchial asthma. ^&^Panic disorder, social phobia, agoraphobia, personality disorder, and psychotic disorder. A multiple regression model was used to estimate the p-value. H/O: history of, NCD: noncommunicable disease, GAD: generalized anxiety disorder, Y-BOCS: Yale-Brown Obsessive Compulsive Scale.

Homocysteine (n = 300)			
Independent observations		β-coff (95% CI)	p-value
Age, years	N/A	-0.01 (-0.16, 0.13)	0.889
Sex	Male	Ref. (0)	N/A
	Female	-4.97 (-7.20, -2.74)	<0.001
Educational qualification	Primary	Ref. (0)	N/A
	Secondary	-1.32 (-3.98, 1.34)	0.329
	Graduate	-0.12 (-3.08, 2.84)	0.937
	Postgraduate	-2.39 (-6.39, 1.60)	0.239
Occupation	Unemployed	Ref. (0)	N/A
	Student	1.51 (-1.79, 4.81)	0.369
	Housewife	1.69 (-2.02, 5.40)	0.371
	Service	2.74 (-0.63, 6.11)	0.111
	Others	8.11 (1.64, 14.6)	0.014
Residence	Rural	Ref. (0)	N/A
	Urban	0.73 (-1.30, 2.76)	0.478
Family pattern	Nuclear	Ref. (0)	N/A
	Joined	-0.74 (-2.70, 1.21)	0.456
	Extended	3.05 (-2.33, 8.42)	0.266
Birth order	First	Ref. (0)	N/A
	Last	0.64 (-1.89, 3.17)	0.621
	Others	0.26 (-1.84, 2.36)	0.810
Family H/O mental illness	No	Ref. (0)	N/A
	Yes	-0.14 (-2.12, 1.83)	0.886
Comorbidity	No comorbidity	Ref. (0)	N/A
	NCD^$^	1.08 (-1.74, 3.89)	0.452
	Skin disorder	3.46 (-1.19, 8.12)	0.144
	Others	-2.68 (-6.67, 1.30)	0.185
Comorbid psychiatric illness	No psychiatric illness	Ref. (0)	N/A
	Depressive disorder	-1.56 (-4.48, 1.35)	0.292
	GAD	-0.18 (-3.04 2.67)	0.899
	Others^&^	-1.45 (-4.84, 1.93)	0.399
H/O substance abuse	No substance use	Ref. (0)	N/A
	Substance use	0.89 (-1.73, 3.51)	0.504
H/O deliberate self-harm	No	Ref. (0)	N/A
	Yes	2.13 (-1.39, 5.65)	0.234
H/O suicide attempts	No	Ref. (0)	N/A
	Yes	-0.78 (-4.67, 3.10)	0.691
Onset	Juvenile (before 18 years of age)	Ref. (0)	N/A
	Adult	-0.30 (-2.64, 2.03)	0.798
Duration of illness	<5 years	Ref. (0)	N/A
	6-10 years	-1.41 (-3.74, 0.93)	0.237
	11-15 years	-2.76 (-5.86, 0.34)	0.061
	>16 years	4.32 (0.29, 8.92)	0.042
Y-BOCS score category	Mild (8-15)	Ref. (0)	N/A
	Moderate (16-23)	1.54 (-1.37, 4.45)	0.298
	Severe (24-31)	2.33 (-0.58, 5.23)	0.116
	Extreme (32-40)	4.04 (0.50, 7.58)	0.026
Insight levels	Poor	Ref. (0)	N/A
	Excellent	2.50 (-0.90, 5.91)	0.149
	Good	0.47 (-2.05, 3.00)	0.713
	Fair	0.30 (-2.70, 3.31)	0.843

**Figure 3:**
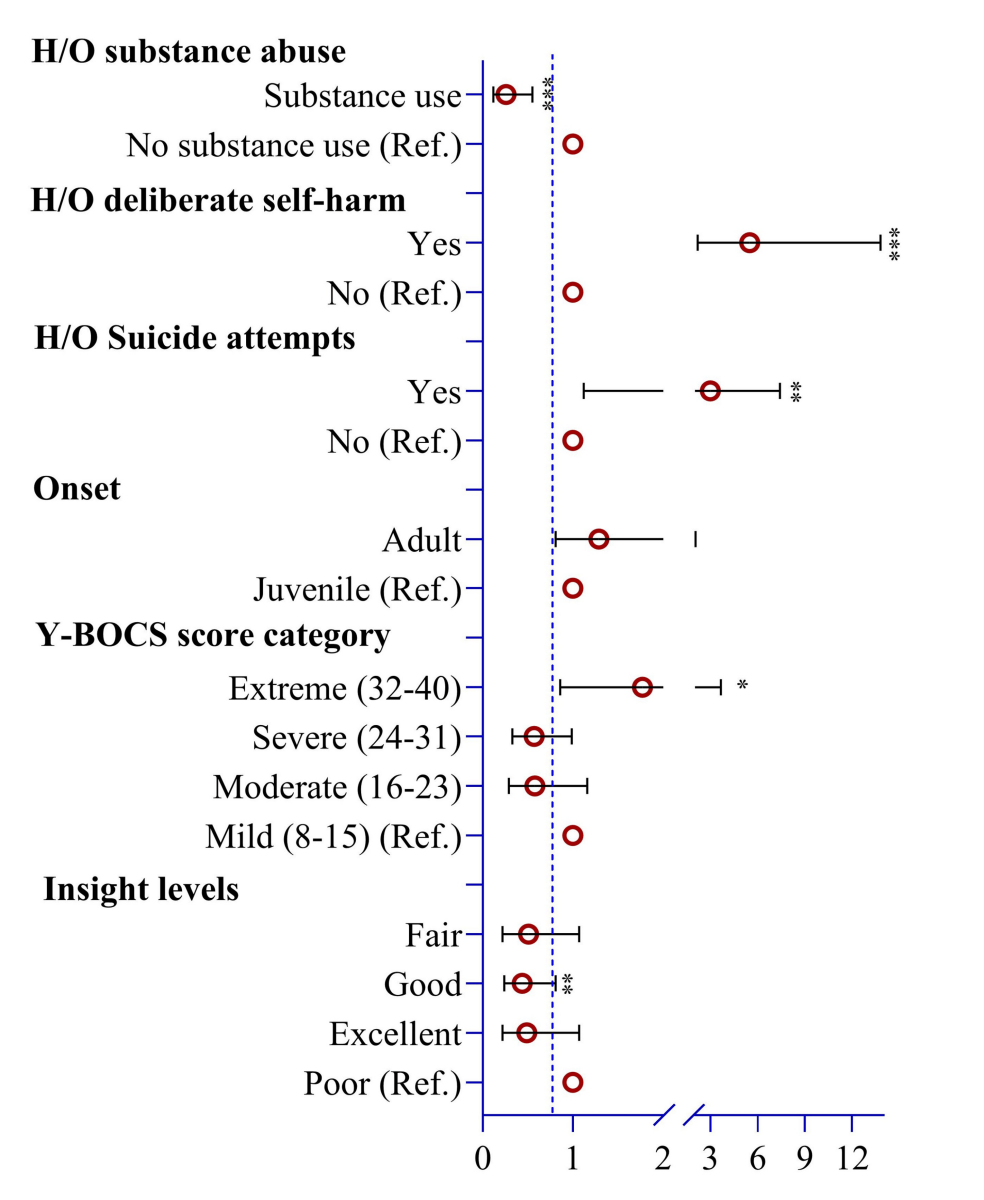
Risks of obsessive-compulsive disorder-related markers among men and women. Note: To estimate the odds ratio and p-value, we used ordinal logistic regression for binary observations and multinomial logistic regression for ordinal observations. H/O: history of, Y-BOCS: Yale-Brown Obsessive Compulsive Scale. Illustration Credit: Md. Ahsanul Haq.

Women exhibited a lower odds ratio (OR) for substance use compared to men (OR = 0.26; 95% CI = 0.12, 0.55; p < 0.001) (Figure 3). Conversely, the likelihood of a history of deliberate self-harm was 5.50 times higher among women (p < 0.001) compared to men. Notably, the odds of a suicide attempt were three times higher among women (95% CI = 1.12, 7.41; p = 0.019) compared to men. Additionally, women were 0.57 times less likely to have a severe Y-BOCS score (p = 0.049) than men. Similarly, the odds of having good insights were 0.44 times lower among women than those with poor insight levels (Figure 3).

## Discussion

This study examines the demographic, clinical, psychosocial, and biochemical characteristics of patients with OCD. The findings highlight several significant associations, including gender differences [94], low serum vitamin B_12_ [12,94] and folic acid [19,95], and elevated concentrations of homocysteine [12], along with clinical and psychosocial markers of disease burden.

OCD is a complex neuropsychiatric disorder with an etiology that is not yet fully understood. Research has suggested that abnormal changes in serum levels of vitamin B_12_, folic acid, and homocysteine may play a role in the development of OCD [12]. A systematic review and meta-analysis of several case-control studies found a statistically significant lower vitamin B_12_ level and significantly higher homocysteine level in OCD groups, though folic acid level was nonsignificantly lower in these groups [12]. Vitamin B_12_ and folic acid levels were considerably lower in OCD patients than in healthy controls in a case-control study conducted in Turkey comparing 50 OCD patients to 50 controls [96].

According to several studies, total plasma homocysteine appears to be a sensitive indicator of a functional folic acid deficit [67,68]. Numerous cell processes contributing to the methylation production of neurotransmitters and nucleic acids need vitamin B_12 _as a co-substrate [97,98]. Homocysteine's total plasma content can be used to estimate intracellular quantities of vitamin B_12_. Methionine is produced by an enzymatic reaction using 5-methyltetrahydrofolate as the methyl donor group [99,100]. As a result, when intracellular vitamin B_12_ concentration increases, homocysteine plasma concentration decreases [101,102]. To produce methionine, which is involved in several biochemical activities, including the metabolism of monoamine neurotransmitters, homocysteine must be methylated using the active metabolite of vitamin B_12_ [103]. Therefore, a deficit in vitamin B_12 _may affect how those neurotransmitters are produced and operate [26,104,105].

Folate deficiency can lead to elevated homocysteine levels, which may contribute to neuropsychiatric side effects by increasing S-adenosyl-homocysteine (SAM). This can inhibit methylation processes and possibly cause direct toxic effects by activating N-methyl-D-aspartate (NMDA) glutamate receptors [106-108].

Gender differences in the risk of OCD clinical symptoms show distinct patterns [109,110]. Women with OCD were found to be significantly less likely to struggle with substance abuse than men [64,111]. Still, women had a much higher likelihood of engaging in self-harm and attempting suicide [112,113]. Our finding of 15.3% (n = 46) is consistent with lifetime rates of substance use disorders (SUDs) in patients with OCD, which have been reported to range from 4.3% to 62.4% [114]. Although we found no significant results, another study reported that the prevalence of vitamin B_12_ and folic acid insufficiencies was higher in the SUD group than in the healthy control group [11,115,116].

Additionally, 10.3% (n = 31) of the participants in our study had a history of deliberate self-harm, and 8% (n = 24) had a history of suicide attempts. A cross-sectional survey of the International College of OCD found that 7.43% have a history of self-injurious behavior [117]. Another multicenter study reported that 9% of participants had a history of a lifetime suicide attempt [118].

In our analysis, we found that women were over five times more likely to engage in deliberate self-harm (p < 0.001) and three times more likely to attempt suicide (p = 0.019) compared to men. In terms of OCD severity, women were less likely to present with severe symptoms (p = 0.049). However, women were less likely to have good insight into their disease condition (p = 0.009) than those with poor insight, indicating that women with OCD were generally less severe, showing a tendency toward poorer awareness or understanding regarding their psychiatric disorders.

**Figure 4:**
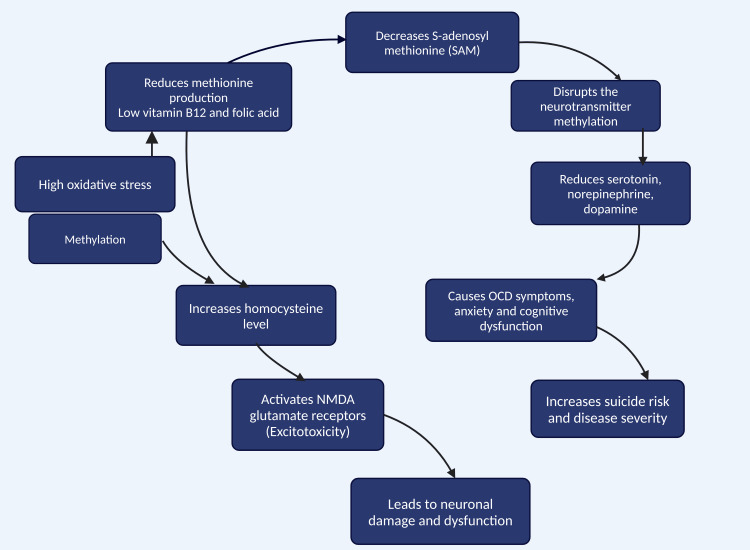
Conceptual model of obsessive-compulsive disorder pathophysiology and biochemical markers. Note: This figure was drawn using the premium version of BioRender (http://BioRender.com/) [86], accessed on February 2, 2025, with agreement license number UG27V4D06B. Illustration Credit: Pratiksha Patel.

Gender differences were statistically significant, with men having higher levels of homocysteine and lower levels of folic acid and vitamin B_12_. In a retrospective study done in Turkey, researchers reported significant folic acid deficiency in male psychiatric patients compared to female patients [119]. Elevated homocysteine levels, particularly among male patients, have been associated with neuropsychiatric conditions and may exacerbate OCD symptoms through increased oxidative stress and impaired methylation processes (Figure 4) [120].

Occupational and psychosocial factors were also associated with biochemical markers concerning OCD [121,122]. Homemakers exhibited lower vitamin B_12_ and higher folic acid levels than unemployed participants, possibly reflecting limited access to a balanced diet or socioeconomic factors [123,124]. Additionally, birth order was identified as a significant factor, with lower vitamin B_12_ levels observed in individuals from higher birth orders [125,126]. The impact of birth order on vitamin B_12_ levels is a novel finding and warrants further exploration. This finding may indicate nutritional disparities in larger families or differences in resource allocation, consistent with previous reports [127]. Although the mechanism underlying this finding is unclear, it may reflect individuals' differential emotional or nutritional environments based on their birth order [128,129]. A significant difference in vitamin B_12_ levels was found between those with and without a family history of mental illness and those with a family history of having lower vitamin B_12 _levels [29,130-133]. A case that Sharma and Biswas reported validated these conclusions [134]. Researchers pointed out hormonal differences, differences in metabolism, and dietary habits within different gender groups as possible reasons behind such findings [135-137].

Participants with NCDs showed lower vitamin B_12_ levels, possibly due to the chronic nature of these conditions, which can interfere with nutrient absorption or utilization [138,139]. NCDs are common in OCD [140]. NCDs, such as hypothyroidism, may cause hyperhomocysteinemia as it lowers the activity of certain liver enzymes that help decrease blood homocysteine levels [141,142]. Numerous investigations have shown that patients with various inflammatory skin conditions have a higher incidence of hyperhomocysteinemia [143,144]. Markers of allergic inflammation were linked to serum folic acid levels [145].

A recent study identified an independent association between low serum folic acid levels (<6.0 ng/mL) and all forms of suicidal behavior. Additionally, another study found that individuals with a family history of suicide had notably lower vitamin B_12 _levels [146]. At the same time, some studies have found no such link [147]. In contrast, our findings showed that patients with a history of deliberate self-harm exhibited significantly higher vitamin B_12_ levels, with an increase of 50.3 mg/L (p = 0.021).

Our findings show that, in addition to women having significantly higher folic acid levels, higher education levels (secondary, graduate, and postgraduate) were associated with increased folic acid levels compared to those with primary education. However, from compulsory school to postgraduate study, OCD is linked to a widespread and significant decline in scholastic attainment, primarily when it manifests early [148]. Educational level considerably influences nutritional food, comprising enough micronutrients and vitamin consumption, which is imperative in improving dietary nutrition quality and quantity [149]. Furthermore, education remains a substantial feature when financial status is considered, impacting healthy nutritional eating and drinking practices [150,151].

Regarding homocysteine, the study found that its levels were significantly higher in patients with a long duration of OCD symptoms and extreme OCD severity, as indicated by the Y-BOCS scores. Elevated homocysteine levels were predominantly observed among male patients and in various occupational groups, aside from the unemployed. The association of longer illness duration (>16 years) with elevated homocysteine levels and extreme symptom severity underscores the chronic nature of OCD and its cumulative impact on metabolic health. High Y-BOCS scores (indicating severe or extreme OCD) were correlated with elevated homocysteine, suggesting a potential role of oxidative stress in exacerbating symptomatology. A study found a negative correlation between patients' folic acid levels and Y-BOCS scores, while a positive correlation was found between homocysteine levels and disease duration. Differences may influence baseline folic acid and homocysteine levels in genetic composition, lifestyle, or dietary status between first-episode, treatment-naïve, and untreated patients and long-term patients [83].

**Figure 5:**
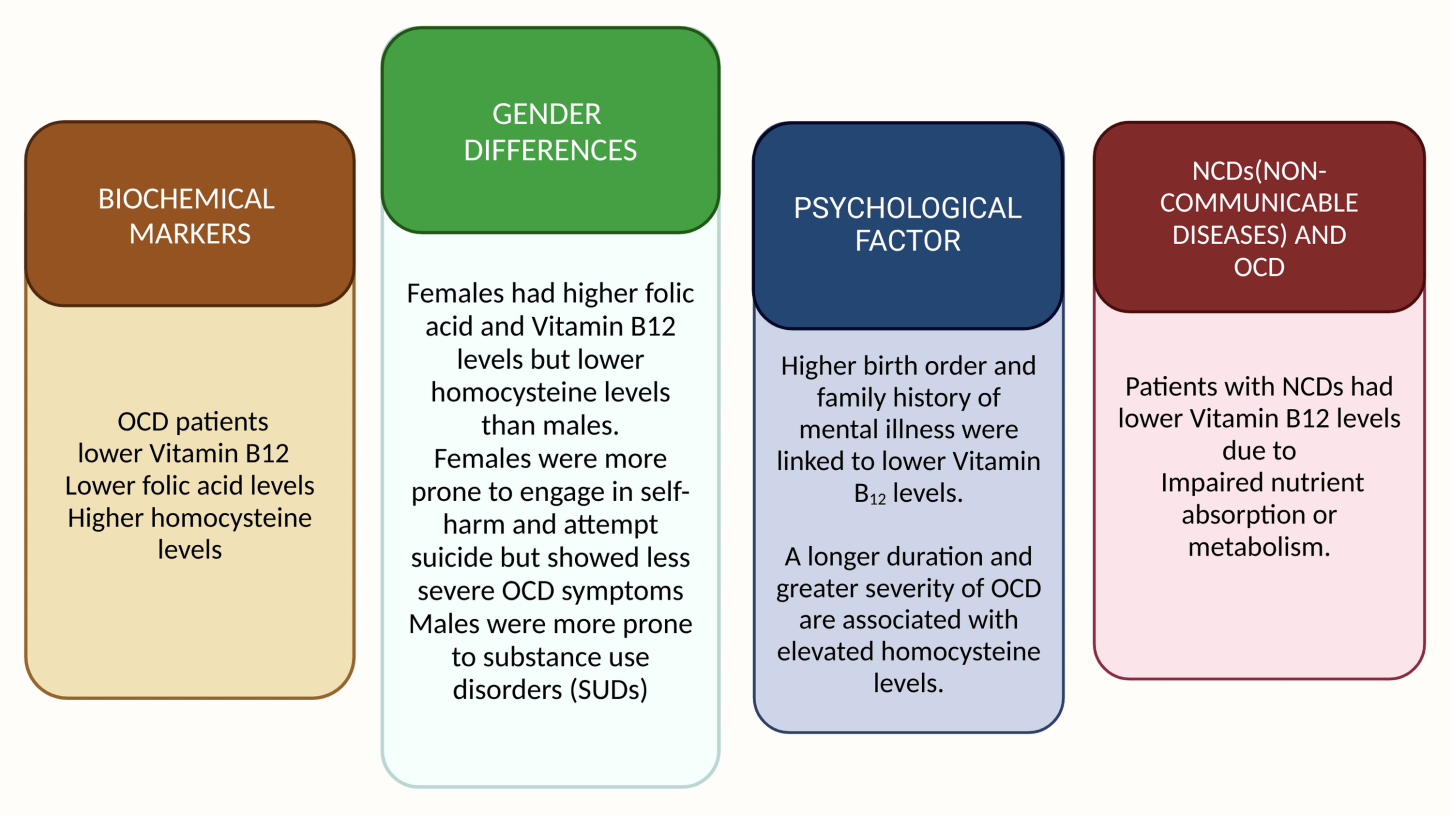
Principal findings of this paper. Note:  This figure was drawn using the premium version of BioRender (http://BioRender.com/) [86], accessed on January 24, with agreement license number DD27TTY29G. Illustration Credit: Pratiksha Patel.

The principal findings of this paper are illustrated in Figure 5.

Limitations of the study

It is crucial to consider the different limitations of the analysis. To begin with, the ability to draw a causal link between OCD and micronutrient levels is limited by the cross-sectional design. Because of the convenience sample approach, the findings might not apply to all OCD sufferers, especially those who are not actively pursuing treatment. Furthermore, this study did not consider environmental factors, such as nutrition and lifestyle, which may influence micronutrient status. Self-reported data on behaviors and comorbidities may be biased and inaccurate. Moreover, the study ignored other possibly relevant micronutrients, focusing solely on vitamin B_12_, folic acid, and homocysteine. Finally, because the sample size was too small for a thorough subgroup analysis, we may not have fully understood the changes in vitamin inadequacies across OCD features. These limitations highlight the need for more studies with a variety of samples and long-term approaches to understand the relationship between micronutrients and OCD better.

Future research recommendations

Future research should focus on well-designed prospective studies with more extensive data size and multicenter that follow micronutrient levels among OCD patients over time, allowing researchers to understand the micronutrient association with the progression of OCD. Intervention experiments, such as randomized controlled trials, that investigate the effects of micronutrient supplementation (e.g., vitamin B_12_ and folic acid) on OCD symptoms would help establish their potential as adjuvant therapy. There should also be a greater emphasis on the necessity of nutritional supplements in psychoeducation sessions.

## Conclusions

Gender differences in OCD patients' serum levels of vitamin B_12_, folic acid, and homocysteine suggest that micronutrient imbalances may influence the disorder's pathogenesis and severity. Male patients had higher homocysteine levels and lower vitamin levels, while female patients showed the opposite, with higher vitamin levels and lower homocysteine. Factors like comorbidities, birth order, and a family history of mental disorders also affect micronutrient levels. Individuals with NCDs and those with a family history of psychiatric conditions generally have lower vitamin levels. More investigation is necessary to validate these associations and prove causation, though, as this could result in better OCD therapies.

## Appendices

Appendix 1 

Semi-structured Questionnaire

Serial number:

Registration number:

Date:        

Phone number:

Patient:

Attendance:

**Table 6:** Questionnaire for unraveling gender differences in obsessive-compulsive disorder: a focus on key micronutrients.

SL	Sociodemographic and clinical status	
1	Name:	Age (in years):
2	Gender	1. Male
		2. Female
3	Marital status	1. Unmarried
		2. Married
		3. Widowed
		4. Separated
		5. Divorced
4	Education	1. Illiterate
		2. Primary
		3. Secondary
		4. Graduate
		5. Postgraduate
5	Occupation	1. Unemployed
		2. Student
		3. Housewife
		4. Service
		5. Business
		6. Others
6	Residence	1. Rural
		2. Urban
7	Religion	1. Muslim
		2. Hindu
		3. Christian
		4. Buddhist
		5. Others
8	Family type	1. Nuclear
		2. Joined
		3. Extended
9	Birth order	1. First
		2. Last
		3. Others
10	Family H/O mental illness (OCD/others)	1. Yes
		2. No
11	Medical comorbidity	1. Hypothyroidism
		2. Diabetes
		3. Hypertension
		4. Skin disorders
		5. Bronchial asthma
		6. Others
		7. None
12	Comorbid psychiatric illness	1. Panic disorder
		2. Social phobia
		3. Agoraphobia
		4. Depressive disorder
		5. General anxiety disorder
		6. Personality disorder
		7. Psychotic disorder
		8. Others
		9. None
13	H/O substance abuse	1. Sedatives
		2. Cannabis
		3. Alcohol
		4. Nicotine
		5. Energy drinks
		6. Others
		7. None
14	H/O deliberate self-harm	1. Yes
		2. No
15	H/O suicide attempts	1. Yes
		2. No
16	Onset	1. Juvenile (before 18 years of age)
		2. Adult
17	Duration of illness	1. <5 years
		2. 6-10 years
		3. 11-15 years
		4. 16-20 years
		5. >20 years
18	Y-BOCS score category	1. Subclinical (0-7)
		2. Mild (8-15)
		3. Moderate (16-23)
		4. Severe (24-31)
		5. Extreme (32-40)
19	DOCS dimensions	1. Dimension 1
		2. Dimension 2
		3. Dimension 3
		4. Dimension 4
20	Insight levels	1. Excellent
		2. Good
		3. Fair
		4. Poor
		5. Lack of insight
21	Level of micronutrients	1. Serum folic acid:
		2. Serum vitamin B_12_:
		3. Homocysteine:
